# Application of Image Sensors to Detect and Locate Electrical Discharges: A Review

**DOI:** 10.3390/s22155886

**Published:** 2022-08-06

**Authors:** Jordi-Roger Riba

**Affiliations:** Electrical Engineering Department, Universitat Politècnica de Catalunya, Rambla Sant Nebridi 22, 08222 Terrassa, Spain; jordi.riba-ruiz@upc.edu; Tel.: +34-937-398-365

**Keywords:** corona discharges, high-voltage applications, visible, ultraviolet, CMOS, detection

## Abstract

Today, there are many attempts to introduce the Internet of Things (IoT) in high-voltage systems, where partial discharges are a focus of concern since they degrade the insulation. The idea is to detect such discharges at a very early stage so that corrective actions can be taken before major damage is produced. Electronic image sensors are traditionally based on charge-coupled devices (CCDs) and, next, on complementary metal oxide semiconductor (CMOS) devices. This paper performs a review and analysis of state-of-the-art image sensors for detecting, locating, and quantifying partial discharges in insulation systems and, in particular, corona discharges since it is an area with an important potential for expansion due to the important consequences of discharges and the complexity of their detection. The paper also discusses the recent progress, as well as the research needs and the challenges to be faced, in applying image sensors in this area. Although many of the cited research works focused on high-voltage applications, partial discharges can also occur in medium- and low-voltage applications. Thus, the potential applications that could potentially benefit from the introduction of image sensors to detect electrical discharges include power substations, buried power cables, overhead power lines, and automotive applications, among others.

## 1. Introduction

Insulation systems withstanding high electric stress are more prone to the harmful effects of electrical discharges. Although partial discharges are typical of high-voltage applications, they also can appear in medium- and low-voltage systems [1]. The IEC 60270 international standard [2] defines partial discharges (PDs) as electrically localized discharges that partially bridge the insulation existing between two conductors. PDs are typical of high-voltage systems, which occur in inhomogeneous sites in insulation systems; either in gases, liquids, or solids [3]; at interfaces and at surfaces; or between a conductor and a metal element not connected to the ground or the high-voltage conductor [4]. PDs are important aging factors in insulation systems [5,6,7] because PD long-term effects include degradation of the electrical characteristics of organic insulation systems [3], thus causing mechanical, thermal, chemical, and structural [8] changes, as well as environmental aging [9]. To ensure the health of insulation systems, PD sources must be identified so that corrective actions can be applied before breakdown occurs [10]. PDs induce a current pulse in the circuit, producing light, an acoustic signal, or a local temperature rise, among others [6]. The measurement of partial discharges (PDs) is a well-established diagnostic method for electrical insulation systems. PDs can be detected by electrical, optical, or acoustic methods, among others, although most of these methods do not allow a direct location of the discharge site or require multiple sensors and complex processing of the information provided by such sensors. PDs and corona discharges are usually detected utilizing specific instruments, including PD detectors, radio interference voltage detectors [11], acoustic sensors [12], optical spectrophotometers [13], spectrometers [14], or radio frequency, VHF, and UHF sensors [15]. However, in general, these instruments do not allow a direct and accurate localization of the discharge site. The optical method is based on detecting the light produced by the discharge [6] as a consequence of different excitation, ionization, and recombination processes occurring during the discharge [16].

A corona is a type of PD occurring in a gas under a highly inhomogeneous electric field [17], which appears at lower voltage than that required for complete breakdown [18]. A corona produces a luminous discharge that forms when the electric field strength around an electrode exceeds a certain critical value [19,20,21]. However, in the presence of an uniform electric field, when increasing the voltage, a complete breakdown of the gap is produced without previous corona activity [22]. The light spectrum of a corona discharge depends on the composition of the surrounding medium and the intensity of the discharge, which depends on factors such as pressure, temperature or others [3]. PDs and corona discharges are greatly affected by the ambient pressure, so low-pressure environments favor corona appearances at lower levels of the applied voltage [23], and at higher pressures, corona discharges weaken [24].

Although PD detection has been a field traditionally dominated by high-voltage engineers, today the situation is changing. It is known that low-voltage applications incorporating inverter-fed loads are subjected to repetitive, high-frequency impulse voltage with short rise times due to high dv/dt rates, thus promoting PD occurrence. Thus, PDs also occur in low-voltage applications in motors [25], cables [1], or automotive applications [26,27], where DC link voltages are increasing, and fast-switching semiconductors are widely applied. However, there are very few experiences for detecting PDs and corona discharges using image sensors, and this is a field allowing for huge expansion in the coming years.

Conventional PD detection systems are mainly based on partial discharge detectors and radio interference voltage measurements. However, emerging methods include ultra-high-frequency (UHF) and acoustic methods, although most recent developments have focused on optical methods [28]. Consequently, new types of solid-state devices are found on the market with improved photon detection efficiency (PDE), some of which are single-photon-sensitive devices [29]. Research on solid-state, solar-blind UV sensors has rapidly matured, offering solar-blind, small-sized, and low-cost sensors [30]. However, most of the abovementioned methods make it difficult to locate the discharge sites, are too bulky and expensive, or the power consumption is too high to be used in emerging needs that require solutions compatible with the Internet of Things (IoT).

In order to detect and locate corona discharges in their early stages, optical methods are advantageous over other technologies due to their relative immunity to electromagnetic and acoustic noise, especially in noisy environments such as power substations or electric aircrafts, among others. Optical sensors include a broad range of optoelectronic devices that produce electrical outputs intended to detect the intensity of light, being sensitive in different bands of the light spectrum, and with different levels of sensitivity, some of which allow the detection of single-photon events [31]. Digital image sensors offer a simple solution to locating discharge sites [32]. Computer vision methods based on machine-learning algorithms have been developed to detect and classify surface discharges in insulators [33,34,35]. Digital image sensors are widely found in digital cameras, smartphones, and many other devices that capture images. When a photon of adequate energy hits a pixel of the sensor, photoelectrons are generated in a number proportional to the intensity of the incident light so that the image can be reconstructed [36]. Each pixel structure has a photosensitive element, usually a photodiode that generates a charge in response to incident light. Charge-coupled devices (CCDs) and complementary metal oxide semiconductor (CMOS) devices are the two main commercial technologies that have traditionally been used as image sensors [37,38,39,40]. Currently available image sensors include a large number of pixels arranged in a matrix of rows and columns, which largely determines the resolution of the image sensor, along with other variables. They can also be used to generate three-dimensional images by applying time of flight (TOF)-based approaches, stereo vision, or the use of structured light [41]. In [42], different methods for changing the spectral responses of photodetectors for CMOS technology without the use of extra processing steps or masks were reported. In [43], on-chip optical photodetectors fabricated using a standard CMOS process and their light-to-frequency converters for readout were presented, while in [44], a similar approach was applied for biological fluid analysis.

Nowadays, a great effort is being made to introduce the IoT in many sectors, which is possible thanks to the incessant improvement in sensor technology and low-cost communication systems, so the detection of electrical discharges should benefit from these advances [45]. Due to the widespread adoption of the IoT, there are many applications that are amenable to incorporating imaging sensors to detect electrical discharges in their incipient stages before significant insulation degradation occurs, which can potentially increase the system reliability and availability and facilitate the application of predictive maintenance tasks. Such applications include overhead power lines, buried power cables, electrical substations, and electric vehicles, among others, because partial discharges can appear in high-, medium-, and low-voltage applications [1].

This work analyzes and reviews state-of-the-art sensors and discusses recent progress, as well as detects the research needs and challenges related to image sensors for detecting and locating partial discharges and, in particular, corona discharges. It is a subject with great potential for expansion, but due to its particular complexity, the introduction of the IoT in this area is still at an early stage. The information presented in this research work is compiled mainly from the latest scientific and technical publications, including journal articles, conference papers, doctoral theses, white papers, and technical reports, among others.

## 2. Sunlight Spectrum and the Solar-Blind Region

Sunlight has a spectrum close to that of a black body at about 5250 °C, which is roughly the temperature at the surface of the Sun. Thus, solar radiation falls within a wide range on the electromagnetic spectrum, including infrared (IR), visible light, and ultraviolet (UV) radiation. Solar radiation also includes shorter-wavelength-ionizing radiation and longer-wavelength radiation, such as radiofrequency and microwaves [1]. However, gases present in the atmosphere have specific absorption bands, so sunlight at sea level has a different spectrum than that outside the atmosphere.

**Figure 1:**
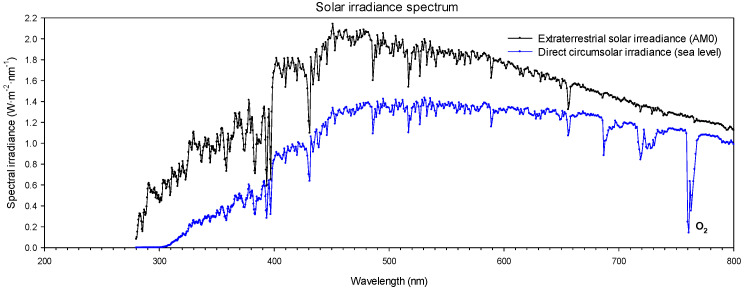
Extraterrestrial solar irradiance (outside the Earth’s atmosphere) and direct circumsolar irradiance spectral data [47] at sea level based on historical data from 1987 to 2003 within the spectral range of 200–800 nm. It shows the O_2_ absorption band.

The visible spectrum falls approximately in the 380–800 nm range, although it is difficult to establish the precise limits of this spectral range because they depend on various factors, such as the responsiveness of the observer or the amount of radiant energy that reaches the retina [46]. UV radiation falls within the spectral range of 100–400 nm, which is further divided into the UVC, UVB, and UVA spectral regions, which include the spectral intervals of 100–280 nm, 280–315 nm, and 315–400 nm, respectively. It is well-known that the stratospheric ozone absorbs UVC and most extraterrestrial UVB radiation. Therefore, solar radiation at the Earth’s surface has wavelengths longer than 280 nm, as shown in Figure 1.

The solar-blind UV band comprises wavelengths in the spectral range of 240–280 nm [48] because, in this spectral range, there is no presence of solar radiation on the Earth’s surface since it is absorbed by the ozone layer. Thus, the main advantage of using solar-blind UV detectors is that they are not interfered with by background solar radiation. Although corona emissions within the solar-blind spectral range are much weaker than within the UVB and UVA regions, it is possible to obtain high-contrast UV images by combining a high signal-to-background ratio with a truly solar-blind filter because it blocks solar radiation outside the band-pass range [49].

Optical sensors that are only sensitive to wavelengths below 280 nm are identified as solar-blind [50], thus being insensitive to the solar radiation wavelengths found at the Earth’s surface. It is worth noting that the concept of solar blindness changes in outer space due to the absence of an atmosphere between the sensor and the Sun [51].

## 3. Corona and Sustained Spark Discharges

As already explained, the spectral composition of the light emitted by an electrical discharge depends on several factors, such as the nature of the gas surrounding the high-voltage electrode or the intensity of the discharge [3].

**Figure 2:**
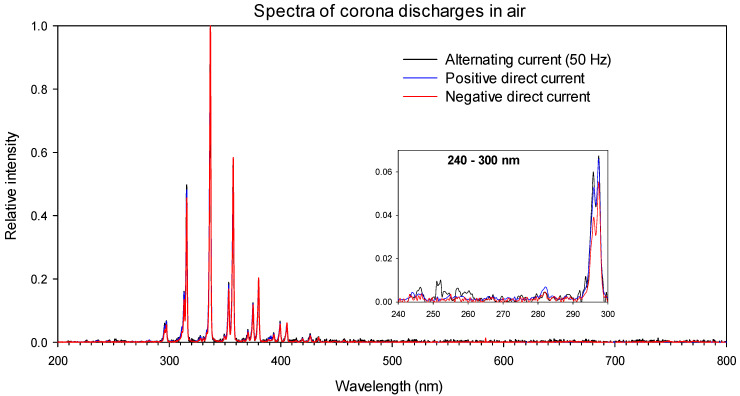
Corona spectra of a needle-plane gap in air.

Figure 2 shows the light spectra of corona discharges in a needle-plane air gap acquired with a Hamamatsu C10082CAH mini-spectrometer, equipped with an A9762-01 UV-visible optical fiber. The spectra were recorded at the AMBER Laboratory of the Universitat Politècnica de Catalunya for this work. As seen, the characteristic wavelengths of the corona in air were mainly within 280 and 420 nm, so they mostly fell within the UV spectrum. This figure also shows that the spectral content of the light emitted by corona discharges was almost independent of the type of supply, that is, alternating current or positive–negative direct current. Figure 2 also shows that the corona discharges had low energy in the UVC spectral region, so to detect such discharges, image sensors that were highly sensitive to the UVC region were required to minimize interferences from sunlight.

**Figure 3:**
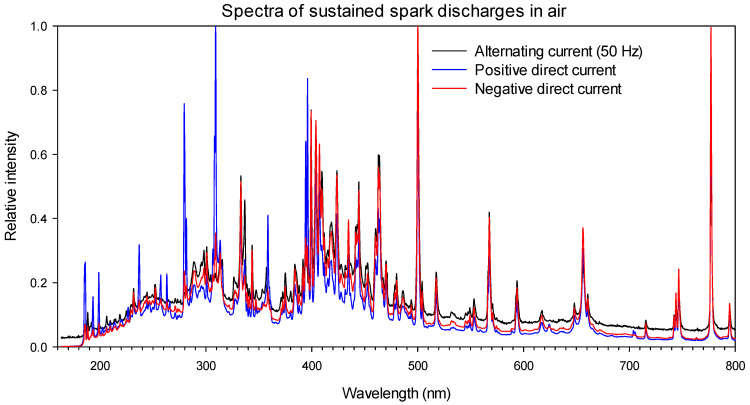
Sustained spark discharge spectra of a needle-plane gap in air.

However, in the case of a sustained spark discharge (electric arc), the spectra differed from those shown in Figure 2, with more components present in the visible region, as shown in Figure 3.

Figure 3 shows the light spectra of a sustained spark discharge (electric arc) in a needle-plane air gap acquired for this work with the same equipment used in Figure 2 at the AMBER Laboratory of the Universitat Politècnica de Catalunya. The results presented in Figure 3 clearly show that the spectra of the sustained spark discharges were more energetic and covered a wider spectral range within both the UV and visible regions.

As seen in Figure 3, a complete spark discharge in atmospheric air emitted light between below 200 nm and 800 nm, thus emitting more visible spectral components compared to corona discharges. It is worth noting that the spectra of discharges occurring on the surface of solid dielectrics are more complex and depend on different factors, including the composition of the solid material, the condition of the surface, and the composition of the gases [6].

## 4. Charge-Coupled Devices (CCDs) and Complementary Metal Oxide Semiconductor (CMOS) Image Sensors

This section details the characteristics of CCDs and CMOS image sensors because they can be used to detect and locate corona discharges.

CCDs and CMOS image sensors have traditionally been the two main commercial technologies for image sensors [37]. For many years, charge-coupled devices (CCDs), which were commercialized in the early 1970s [52], have been the most widely used imaging sensors for digital cameras. CCD detectors have good spatial resolution and linearity, as well as high quantum efficiency (QE) over a wide range of wavelengths, from gamma rays to far-infrared [53], and have a low noise level. QE is defined as the ratio between the number of generated photoelectrons and the number of incident photons in a photodetector. It reflects the photon sensitivity of a photodetector as a function of the wavelength of the incident photons, thus greatly impacting the generated photocurrent [54]. In CMOS sensors, the QE can be improved using an appropriate arrangement of dielectric layers on top of the photodiode surface because they act as a thin-film interference filter, thus influencing the optical transmittance at each wavelength [44].

Because of their appealing features, CCDs have been quickly applied by engineers and physicists for most UV, visible, and IR applications. However, one of the disadvantages of CCDs over other image sensors is their high cost [55]. In [53], it was shown that back-illuminated CCD sensors were sensitive below 200 nm, with a QE of 50% at about 40 nm and 10% at 200 nm. Therefore, backside illumination is often preferred for UV imaging [56]. CCDs are also used to detect gamma rays. In [57], a CsI scintillator was used to convert the incident gamma rays into visible light photons, which were collected with a CCD image sensor.

Today, CCDs have almost been replaced by CMOS sensors, because CMOS technology offers substantial advantages, such as low voltage supply, low power consumption, and longer battery life, as well as integration, thus allowing for the manufacture of single-chip miniaturized digital cameras or high-speed imaging [58]. CCD sensors offer some advantages over CMOS sensors; they tend to suffer from less sources of noise [59] and allow high-quality photographs [60]. Initially, CMOS sensors had poor pixel performance compared to CCD imagers, but they have evolved to achieve high-quality image performance, which is now comparable to that of CCDs [61].

Today, CMOS image sensors are widely used in consumer electronics digital cameras, including smartphone cameras and digital single-lens reflex (DSLR) cameras [32,62]. In particular, back-illuminated CMOS sensors have some advantages over conventional CMOS sensors since back-illuminated sensors have the circuitry to transmit the electronic signals generated in the pixels at the back of the sensor instead of the front. As a result, back-illuminated sensors capture more light, thereby improving low-light performance and QE at wavelengths from UV [56] to IR [63].

Both CMOS and CCD imagers offer excellent quantum efficiency within the visible range, that is, more than 50% in the 400–700 nm spectral range, although the QE is more limited within the UV range [64].

With the constant improvement in digital camera technology, DSLR cameras make it possible to capture coronal activity at long exposure values, preferably when the environment is darker than the light emitted by the corona. Thus, by adjusting different parameters (exposure time, aperture, and ISO sensitivity), the light from the corona can be detected [65].

Due to their high quantum efficiency, low noise, and low dark current, most CMOS and CCD imaging sensors incorporate pinned photodiodes as the main structure of the photodetector [52], and thus, photodiode-based sensors exhibit enhanced performances [64]. The incident irradiation on the photodiode is converted into a small electric signal, whose magnitude depends on the intensity of the incident irradiation and the spectral sensitivity of the photodiode [66]. Both CCD sensors and CMOS image sensors are made up of an array of pixels or light-sensitive elements. They convert the incident irradiation into a charge, i.e., photoelectrons [67], due to the photoelectric effect. However, CCD and CMOS technologies treat the generated charges differently.

**Figure 4:**
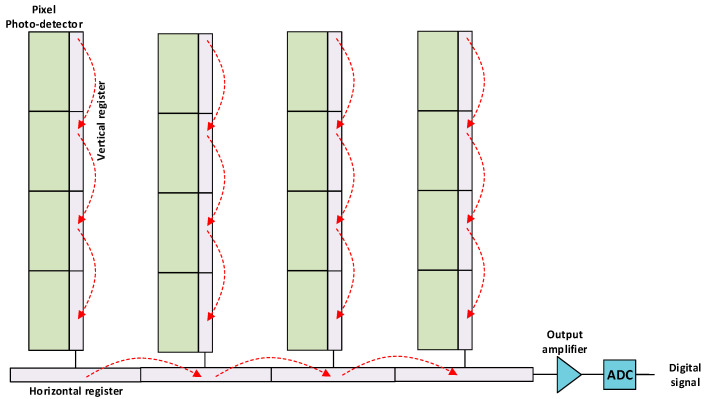
Schematics of a CCD image sensor.

In CCD image sensors, the weak electrical signal generated by each pixel moves through the circuit via vertical and horizontal shift registers, which convert the charge to voltage. The light level is then sampled in the readout circuitry, which includes an amplifier and an analog-to-digital converter (ADC). Therefore, the electrical signal generated by each individual pixel must move around the array, as seen in Figure 4, which requires power and time because the register content is read out serially.

**Figure 5:**
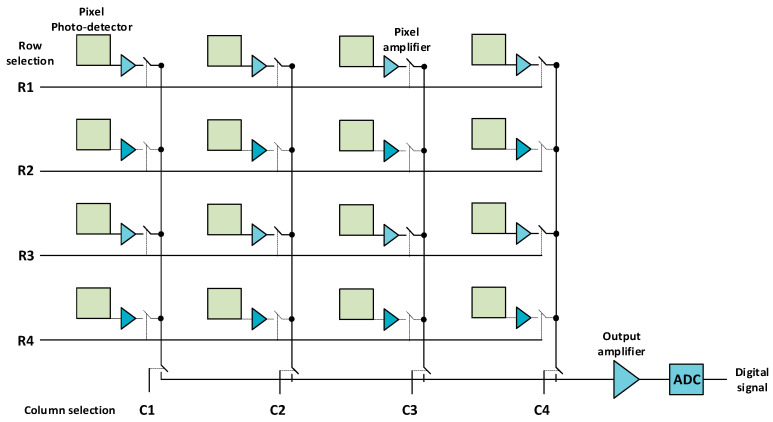
Schematics of a CMOS image sensor.

In contrast, in CMOS sensors, each pixel includes its own amplifier, and each column of pixels is processed simultaneously through an ADC. Thus, the charge generated by each pixel is directly converted into voltage by the amplifier included in each pixel and is sent to the ADC. Thus, as seen in Figure 5, the responses of all the pixels in a column can be read simultaneously in a parallel configuration, making it suitable for fast image acquisition. CMOS image sensors can also be manufactured using semiconductors other than silicon, so they are sensitive to wavelengths other than the visible spectrum.

### Front-Illuminated versus Back-Illuminated Image Sensors

The pixels include the photodiodes or light-to-charge conversion elements. The pixels are interconnected with metal wires, forming a matrix so that incoming light can be spatially localized.

In both CCD and CMOS image sensors, the size of the array of pixels influences the signal-to-noise ratio and the dynamic range of the sensor, which is related to the maximum achievable signal divided by the noise of the camera, which contributes to the quality of the image [68].

**Figure 6:**
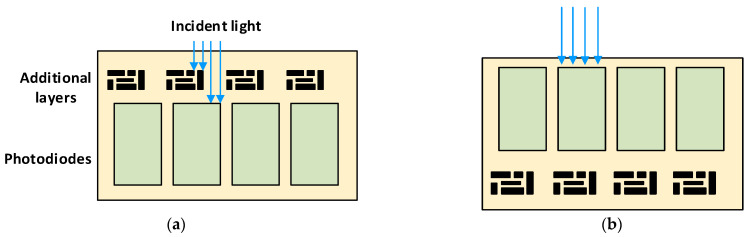
Schematics of (**a**) a front-illuminated image sensor and (**b**) a back-illuminated image sensor.

Back-illuminated image sensors are more sensitive than front-illuminated sensors because back-illuminated sensors have a higher fill factor, that is, the ratio of the effective light-sensitive area to the total area of a pixel [58], so image sensors with higher fill factors tend to be more sensitive. For this reason, front-illuminated sensors have additional layers of wiring, transistors, and capacitors on top of the photodiode. These additional layers tend to scatter and absorb incident light, resulting in a poorer light-to-charge conversion factor. This issue is overcome by the use of back-illuminated sensors, where the additional layers are built on the side opposite to the incoming light, as shown in Figure 6, so that the incident light encounters fewer layers above the photodiodes.

## 5. Ultraviolet (UV) and Visible Imaging Applied to Corona Discharges

It is well-known that air molecules in the vicinity of a high-voltage electrode become ionized when the electric field exceeds the inception threshold value [69]. During the ionization process typical of an electrical discharge, the electrons released change their energy levels, thus emitting ultraviolet and visible photons, broadband electromagnetic waves, and acoustic emissions, as well as generating chemical components such as traces of nitric acid or ozone, among others [70]. In [13,71,72,73] the UV and visible spectra of electrical discharges have been presented, showing that they cover the 200–700 nm range. It is known that measurements using digital cameras can be of great help in detecting part of the UV spectrum [74].

UV imaging was developed to detect the UV signals produced during the discharge process, which is widely applied for live detection [75]. UV imaging enables the source of the discharge to be detected and located, as well as its intensity after appropriate data processing and overlapping with visible images. Immunity to electromagnetic interference is a key advantage of UV-based methods over classical electrical methods, especially in real operating environments where electromagnetic interference is an issue [76].

In [77], it was concluded that the 357.6 nm Fraunhofer line could be used to detect daytime corona activity by applying an extremely narrow optical band-pass filter and additional suitable signal processing. Optical filters made of thin-film interference layers, Fabry–Perot etalons, etc., play a key role in image sensors, as well as in spectrophotometric and colorimetric measurements [43,44,78]. However, as explained, measurements in daylight conditions are challenging for UV and visible detectors due to interference from sunlight. To avoid this problem, UV solar-blind imaging sensors and standard visible imaging sensors can be combined in a single unit to detect, locate, and quantify electrical discharges, even in daylight conditions, so that different types of corona cameras are commercially available [49,79,80,81]. To ensure free transmission within the near-UV spectral range, digital cameras must be equipped with quartz lenses [82,83].

### 5.1. Quantification of the Intensity of Corona Discharges

In addition to being able to detect and locate corona discharges, it is also very interesting to quantify the impact of the discharges, that is, to determine their intensity. An early study found that there was a correlation between the light intensity of corona images obtained with a daylight camera and the discharge magnitude determined with conventional partial discharge equipment [84]. In [85], it was found that, by using pixel saturation of digital visible images of corona discharges, it was possible to assess the degree of degradation of polymeric insulation samples. Different studies have revealed that the number of photons released by discharges depends upon their energy [77,86,87]. This quantification is of special interest in order to apply predictive maintenance plans since, by studying the intensity of discharges, it is possible to determine their evolution, so it is possible to develop computer tools to predict the expected time to failure. This is an open field that lacks experimental works and experience.

Through the use of visible and UV imaging sensors, it is not only possible to detect and locate the origin of discharges, but also to determine their intensity. In [76], it was shown that the number of photons per unit time detected by a UV camera fit well with the level of corona discharge and the magnitude of the corona current. In [87], it was also shown that the energy contained in a visible image of an electrical discharge was proportional to the electric energy involved in the discharge process. This corroborated a direct correlation between the energy released by the physical process itself and the energy level impressed in corona images. Similar conclusions have been reached in [65,86]. Therefore, it has been concluded that, through the proper processing of acquired images, it is possible to determine the intensity of corona discharges.

In [11], it was shown that the sensitivity of image sensors was almost the same as that of very sensitive sensors, such as RF antennas and current leakage sensors, to detect corona discharges at a very early stage. In [88], it was shown that, in an unscreened laboratory, digital image sensors offered similar sensitivity to that of commercial PD detectors (one of the most sensitive instruments) but at a much lower cost, while allowing the easy and accurate location of discharge sites at an initial stage.

### 5.2. Use of CCD Imagers to Detect, Locate, and Quantify Corona Discharges

CCD imagers have been and still are applied to detect corona discharges. This section reviews some of the information found in the scientific literature. In [89], a CCD camera was used to capture images of tracer particles to study the electrohydrodynamic (EHD) flow generated by corona discharges produced in an electrostatic precipitator. In [90], the spatial distribution of air temperatures due to corona discharges was obtained using a CCD camera. In [91], a CCD camera covering the 190–1100 nm spectral range was applied to detect UV emissions from corona discharges produced by overhead power lines in the range from 250 nm to 400 nm. In [92], a CCD imager was used to detect partial discharges in transformer oil. In [84], it was shown that there was a correlation between a visual image acquired with a CCD imager and the magnitude of the corona discharge measured with conventional partial discharge equipment. In [93], a solution to detect corona discharges based on two CCDs imagers was proposed. While one sensor was sensitive to the visible spectrum, the other was sensitive to the UV spectrum. The two sensors received the same image captured by the lenses, which passed through a beam splitter. Next, a UV filter and an opaque solar filter respectively filtered the two images in parallel. The first image excited the visible CCD, while the second one excited the UV CCD. Finally, the two outputs of the CCDs were processed and merged into a single image. This is the principle used in most commercial solar-blind corona cameras [49,79,80,81], which are specially designed to detect, locate, and quantify corona discharges.

### 5.3. Use of CMOS Imagers to Detect, Locate, and Quantify Corona Discharges

CMOS image sensors have also been widely used to detect and locate corona discharges; some of the information found in the literature is reviewed in this section. Similar to [93], in [94] it was proposed to use a dual-spectra camera sensor to detect corona discharges. It included a low-light CMOS imager using a visible light channel and a UV imager. While the UV sensor allowed the location of the discharges, the CMOS sensor enriched the details of the image and helped to locate the discharges in a complex background. A real-time dual-UV-visible imager for outdoor environments was presented in [95]. From the top, the device included, in order, a protecting film, a top transparent electrode, a very thin organic photoconductive film (OPF) imager, and finally, a bottom pixel electrode, which was connected to the CMOS circuits. CMOS sensors used in digital cameras are also sensitive to gamma and X-rays [96].

Due to the constant development of digital cameras, the effective number of pixels in a visible image is usually much greater than that found in high-speed, UV, or infrared cameras, and therefore, the image provides more spatial information of the discharge [97]. Different authors have used commercially available CMOS sensors intended for visible photography to detect corona emissions and locate the discharge points [65,98] because CMOS sensors are known to be sensitive in the visible and UV regions. In [32], a Sony IMX219 miniature digital sensor was used to detect a visual corona effect, whose spectral response could be found in [99]. In [100], a visual corona was detected using a 20.2 Mpixels CMOS APS-C sensor. Other studies have captured corona using high-speed digital cameras [101,102,103]. In [88,100,104], unmodified Canon EOS-70D DSLR cameras equipped with 20.2 Mpixels APS-C CMOS sensors have been used to detect and locate the corona sites of high-voltage substation connectors under alternating current supplies, while in [105] the same camera was used to analyze the corona effect under direct current supply of both polarities.

In [106], the authors proposed to use a solar-blind UV optical filter and a Te-Cs cathode to remove light falling outside the solar-blind band, as well as a UV image intensifier coupled with a CMOS image sensor, because this configuration allowed the detection of single-photon events.

### 5.4. Use of Dual-Spectra Cameras to Detect, Locate, and Quantify Corona Discharges

In the last two decades, UV-visible dual-spectra cameras have become popular noncontact methods for the incipient fault diagnosis of overhead power lines due to their sensitivity, noncontact measurements [107,108], and robustness, as well as because they allow the detecting and locating of corona discharge sites [109]. They are also known as daylight UV cameras due to their ability to detect and locate corona sites in daylight conditions for field tests and in laboratory experiments [110]. There are several commercial cameras that combine visible and solar-blind UV detectors. They are mostly used to inspect high-voltage infrastructure [111]. Most of these cameras are based on CCD sensors [112] or CMOS sensors [113] because they have been shown to be sensitive enough to detect weak corona discharges [114], so dual-spectra UV-visible cameras have been extensively used to detect corona discharges [115,116]. It is known that there is a correlation between the observed optical energy and the energy loss due to corona discharges [117]. After adequate processing of the information, dual-spectra cameras allow the intensity of the discharges to be quantified [114,118].

**Figure 7:**
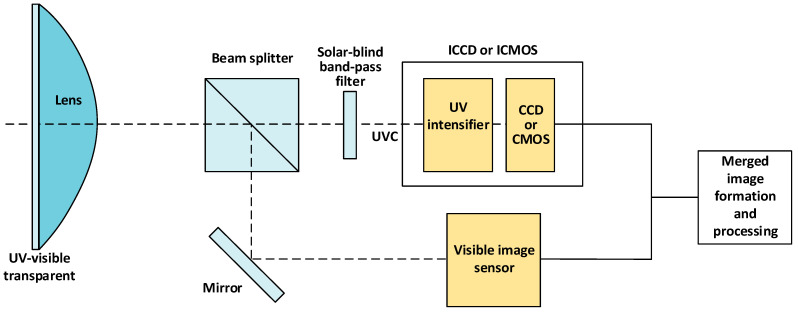
Schematics of UV-visible dual-spectra imager adapted from [109].

Most dual-band cameras use a solar-blind UVC filter combined with an image-intensified CCD (ICCD) [114] or an intensified CMOS (ICMOS) [94,106,117,119], as illustrated in Figure 7.

The camera shown schematically in Figure 7 allows the corona effect to be detected in daylight since it uses image detectors that simultaneously acquire images in the 250–280 nm UVC band and in the visible band. Once both images are superimposed, the discharge sites are located thanks to the UVC image, which is superimposed on the visible light image [120].

The use of UV-visible dual-spectra cameras is well documented in the technical literature to detect discharges in different applications, including insulator strings [108,121,122], transformer bushings [123], motor windings [124], transmission line inspection using unmanned aerial vehicles (UAVs) [125].

There is also experience in developing single-exposure image sensors for the simultaneous acquisition of UV and visible images without using optical filters by employing low- and high-sensitivity pixel types arranged in a checkered pattern, from which the UV and visible images can be generated [126].

### 5.5. Smartphone Image Sensors for Visible and UV Radiation Detection

Due to their low cost and small size, smartphones mostly incorporate CMOS image sensors [60]. In recent years, smartphone image sensors have been widely used to detect UV radiation [127]. In [128,129], the authors each characterized and analyzed the performance of a smartphone camera in the UBA 320–400 nm range. Similarly, in [130] the UVB waveband response of a CMOS smartphone camera was also characterized and analyzed. In [131], it was shown that a smartphone camera could detect 305 nm UVB radiation without replacing the outer lens. Similar results were reported analyzing the 312 nm band in [132].

However, few attempts have been made to detect corona discharges using unmodified smartphone cameras. For example, in [32] a low-cost 8 Mpixels Sony IMX29 back-illuminated CMOS smartphone camera controlled by a Raspberry pi microcontroller was applied to detect corona discharges under low-pressure aeronautic conditions within the 20–100 kPa pressure range. In [11], a 48 Mpixels Sony IMX586 UV-visible image sensor mounted in an unmodified smartphone was used to detect and localize corona discharges in the 10–100 kPa pressure range, along with other sensors. The results showed that the sensitivity of this camera was similar to other state-of-the art sensors. The same image sensor has been used in [45,133,134].

In [96], smartphone cameras were used to detect gamma rays generated by different gamma ray sources (cobalt-60, cesium-137, and iridium-192) because the interactions of such rays with the CMOS sensor produced flashing bright spots superimposed on a dark background.

### 5.6. Visible and UV Images of Corona and Spark Discharges

**Figure 8:**
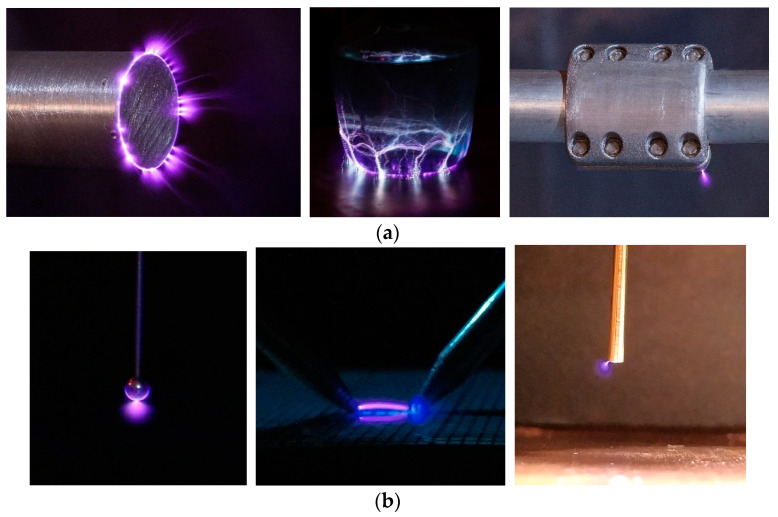

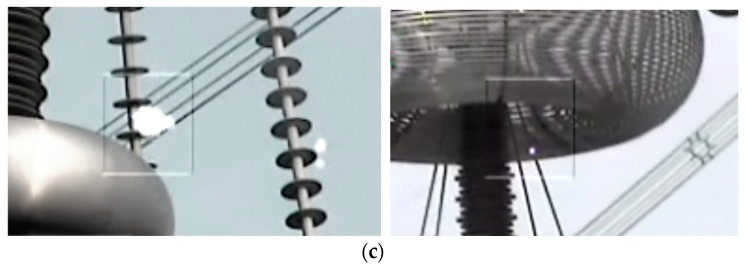
Corona photographs captured with different sensors. (**a**) UV-visible corona photographs captured with a Canon EOS-70D DSLR camera. (**b**) Corona photographs captured with a Sony IMX586 smartphone camera. (**c**) Daylight corona photographs captured with a solar-blind dual-spectra Daycor Superb camera.

This section shows UV-visible and solar-blind UV-visible photographs captured by the author of this work at the facility of AMBER Laboratory of the Universitat Politècnica de Catalunya, where the corona sites were clearly identified. These photographs are summarized in Figure 8.

## 6. Identified Challenges and Research Needs

Having reviewed the state-of-the-art research, this section highlights gaps in the existing body of research and identifies future research directions for corona discharge detection and identification.

In the previous sections, it was shown that image sensors were capable of not only detecting corona discharges, but also of locating the corona sites and quantifying the discharge intensity. Although image sensors have evolved dramatically in the last decades, they still require further improvement to be applied in many applications related to corona detection, location, and quantification, specific those compatible with the IoT, where small sensors with low cost and low power consumption are required.

It is also appealing to develop image sensors for corona detection in daylight conditions in order to develop early fault diagnosis and inspection approaches for power lines, substation assets, and in general, to detect corona discharges in high-, medium- or low-voltage systems due to the limitations they still offer for this specific application, which are described below.

In order to meet the requirements detailed above, the challenges and corresponding research needs that are identified are detailed in the following paragraphs.

Due to interference from sunlight, daylight imaging involves focusing on both the solar-blind band and the visible spectrum. Current UV-visible, solar-blind, dual-band imagers are very expensive, in the order of several tens of thousands of euros. In their early stages, corona discharges emit in the solar-blind band with low intensity, so dual-spectra daylight imagers often use a solar-blind UV filter combined with an image intensifier to amplify the energy in the UV channel. A major challenge is to design specific imagers with very high quantum efficiency in the solar-blind spectral region. This strategy could avoid the use of image intensifiers or photomultipliers, which increase the cost of the entire system and often require high-voltage supply and external cooling, thus increasing the size, weight, cost, and power consumption. Another possibility is to develop single-exposure image sensors for the simultaneous acquisition of UV and visible images without optical filters using approaches inspired by [126] or other possibilities open for research. Therefore, drastic reductions in cost, power consumption, volume, and weight are required, especially to install a network of corona imagers for the automatic inspection of high-, medium-, and low-voltage assets in an IoT-compatible approach.

Due the widespread use of smartphones, the camera has become a central element. Camera sensors for smartphones are incessantly evolving, thus being very attractive for many applications since they feature different features, including high sensitivity, small size, and low cost. Therefore, smartphones are being progressively introduced in many scientific disciplines, such as healthcare [135], agriculture, environment, and food, among others [136]. Despite their widespread use, they are still underutilized for the detection of corona discharges, so this application deserves special attention, especially because of the constant evolution of CMOS image sensors specifically developed for smartphone cameras.

Finally, the possibility to quantify the intensity of discharges is an open field of research, which is especially interesting for applying predictive maintenance plans. There is an urgent need to develop tools to quantify discharges to determine the state of health of insulation, as well as the expected time to failure and the remaining useful life (RUL). Such tools could potentially track the evolution of discharges so that corrective actions could be anticipated, thus facilitating the application of predictive maintenance strategies. This is an open field lacking experimental works and experience.

## 7. Conclusions

Due to the availability of low-cost sensors; low-cost, low-power microprocessors; and the availability of cost-effective and small-sized communication systems, the IoT is being progressively introduced in many technological applications. Thus, there is a need to develop partial discharge and corona detection, location, and quantification image sensors compatible with the IoT so that early insulation faults can be detected and diagnosed. In this way, corrective actions can be applied before major failures occur, thus enabling predictive maintenance approaches to be applied. This paper reviewed the state-of-the-art research in the use of electronic image sensors for detecting, locating, and quantifying corona discharges in air-insulated systems because this area has enormous potential for expansion due to the critical consequences of such electrical discharges and the inherent difficulty in their detection, especially in noisy environments. In particular, the main identified technologies based on image sensors are summarized below:

- CCD image sensors, which offer good spatial resolution and linearity, as well as high quantum efficiency over a wide spectral range from gamma rays to the far-infrared, and present a low noise level;
- CMOS image sensors, which offer substantial advantages, such as lower voltage supply, lower power consumption, longer battery life, and integration, thus allowing the manufacture of single-chip miniaturized digital cameras and high-speed imagers;
- Solar-blind dual-spectra UV-visible cameras based on CCD or CMOS sensors to detect corona discharges, although they are bulky and very expensive;
- Smartphone image sensors, which are increasingly used to detect electrical discharges due to their low cost, small size, and constant technological evolution.

The paper also discussed the recent progress in this area and identified the challenges and research opportunities that need to be addressed to apply image sensors in this area. Traditionally, electrical discharges have been associated with high-voltage applications, but they can also occur in medium- and low-voltage systems. Therefore, there is a wide range of potential applications likely to benefit from the introduction of image sensors to detect electrical discharges. They include buried power cables, overhead power lines, electrical substations, renewable generation, and electrical vehicles, including automobiles, trains, ships, and aircraft, among others.

## Data Availability

Not applicable.

## References

[1] Kim J., Kim K.-I. (2021). Partial Discharge Online Detection for Long-Term Operational Sustainability of On-Site Low Voltage Distribution Network Using CNN Transfer Learning. Sustainability.

[2] International Electrotechnical Commission (2000). IEC 60270:2000 High-Voltage Test Techniques—Partial Discharge Measurements.

[3] Muhr M., Schwarz R. (2009). Experience with optical partial discharge detection. Mater. Sci. Pol..

[4] IEEE Power Engineering Society, Insulated Conductors Committee, Institute of Electrical and Electronics Engineers, IEEE-SA Standards Board (2007). IEEE Guide for Partial Discharge Testing of Shielded Power Cable Systems in a Field Environment.

[5] Rosle N., Muhamad N.A., Rohani M.N.K.H., Jamil M.K.M. (2021). Partial discharges classification methods in xlpe cable: A review. IEEE Access.

[6] Xu Y., Yu M., Cao X., Qiu C., Chen G. (2000). Comparison between optical and electrical methods for partial discharge measurement. Proc. IEEE Int. Conf. Prop. Appl. Dielectr. Mater..

[7] da Costa I.B.V., Weber G.H., Gomes D.F., Galvão J.R., da Silva M.J., Pipa D.R., Ozcáriz A., Zamarreño C.R., Martelli C., Cardozo da Silva J.C. (2020). Electric discharge detection and localization using a distributed optical fiber vibration sensor. Opt. Fiber Technol..

[8] Florkowski M. (2020). Hyperspectral imaging of high voltage insulating materials subjected to partial discharges. Measurement.

[9] Stone G.C. (2005). Partial discharge diagnostics and electrical equipment insulation condition assessment. IEEE Trans. Dielectr. Electr. Insul..

[10] Borghei M., Ghassemi M. Discrimination of Single- and Multi-Source Corona Discharges using Deep Residual Network. Proceedings of the 2021 IEEE Electric Ship Technologies Symposium (ESTS).

[11] Riba J.-R., Gomez-Pau A., Moreno-Eguilaz M. (2020). Sensor Comparison for Corona Discharge Detection Under Low Pressure Conditions. IEEE Sens. J..

[12] Souza A.L., Lopes I.J.S. (2015). Experimental investigation of corona onset in contaminated polymer surfaces. IEEE Trans. Dielectr. Electr. Insul..

[13] Kozioł M., Nagi Ł., Kunicki M., Urbaniec I. (2019). Radiation in the Optical and UHF Range Emitted by Partial Discharges. Energies.

[14] Vieira A.L., Silva T.V., de Sousa F.S.I., Senesi G.S., Júnior D.S., Ferreira E.C., Neto J.A.G. (2018). Determinations of phosphorus in fertilizers by spark discharge-assisted laser-induced breakdown spectroscopy. Microchem. J..

[15] Zachariades C., Shuttleworth R., Giussani R. (2020). A Dual-Slot Barrier Sensor for Partial Discharge Detection in Gas-Insulated Equipment. IEEE Sens. J..

[16] Burjupati N.R., Puhan D.K., Sharma R. Opto Electronic Technique for Detection of Corona Discharges in Air and Oil. Proceedings of the 2019 IEEE Electrical Insulation Conference (EIC).

[17] Borghei M., Ghassemi M. (2022). Separation and Classification of Corona Discharges under Low Pressures Based on Deep Learning Method. IEEE Trans. Dielectr. Electr. Insul..

[18] Anis H., Srivastava K.D. (1982). Pre-breakdown Discharges in Highly Non-uniform Fields in Relation to Gas-insulated Systems. IEEE Trans. Electr. Insul..

[19] Rudd S., McArthur S.D.J., Judd M.D. (2010). A generic knowledge-based approach to the analysis of partial discharge data. IEEE Trans. Dielectr. Electr. Insul..

[20] IEEE (2000). The Authoritative Dictionary of IEEE Standards Terms.

[21] Zhang C., Yi Y., Wang L. (2016). Positive dc corona inception on dielectric-coated stranded conductors in air. IET Sci. Meas. Technol..

[22] Hernández-Guiteras J., Riba J.-R., Casals-Torrens P. (2013). Determination of the corona inception voltage in an extra high voltage substation connector. IEEE Trans. Dielectr. Electr. Insul..

[23] Emersic C., Lowndes R., Cotton I., Rowland S., Freer R. (2017). The effects of pressure and temperature on partial discharge degradation of silicone conformai coatings. IEEE Trans. Dielectr. Electr. Insul..

[24] Sun Y., Chen Y., Ci W., Liang J., Dong J., Wang H., Zhang B. Application of Robot Detection Equipped with Ultraviolet Imaging. Proceedings of the 2021 IEEE 4th Advanced Information Management, Communicates, Electronic and Automation Control Conference (IMCEC).

[25] Ogundiran Y.L., Griffo A., Sundeep S., Gonzalez F.A., Wang J. A Novel Ring-Shaped Fractal Antenna for Partial Discharge Detection. Proceedings of the 2021 IEEE Energy Conversion Congress and Exposition (ECCE).

[26] Benmamas L., Teste P., Krebs G., Odic E., Vangraefschepe F., Hamiti T. Contribution to partial discharge analysis in inverter-fed motor windings for automotive application. Proceedings of the 2017 IEEE Electrical Insulation Conference (EIC).

[27] Driendl N., Pauli F., Hameyer K. (2022). Influence of Ambient Conditions on the Qualification Tests of the Interturn Insulation in Low-Voltage Electrical Machines. IEEE Trans. Ind. Electron..

[28] Sefl O., Prochazka R., Haller R., Monkman G.J. Alternative Approach to Optical Detection of Partial Discharges in Air. Proceedings of the 2021 IEEE Conference on Electrical Insulation and Dielectric Phenomena (CEIDP).

[29] Renker D., Lorenz E. (2009). Advances in solid state photon detectors. J. Instrum..

[30] Sadler B.M., Abou-Galala F., Chen G., Xu Z. (2008). Experimental evaluation of LED-based solar blind NLOS communication links. Opt. Express.

[31] Ren M., Zhou J., Song B., Zhang C., Dong M., Albarracín R. (2017). Towards Optical Partial Discharge Detection with Micro Silicon Photomultipliers. Sensors.

[32] Riba J.-R., Gómez-Pau Á., Moreno-Eguilaz M. (2020). Experimental Study of Visual Corona under Aeronautic Pressure Conditions Using Low-Cost Imaging Sensors. Sensors.

[33] Wang X., Zhang Y. Insulator identification from aerial images using Support Vector Machine with background suppression. Proceedings of the 2016 International Conference on Unmanned Aircraft Systems (ICUAS).

[34] Pernebayeva D., Irmanova A., Sadykova D., Bagheri M., James A. (2019). High voltage outdoor insulator surface condition evaluation using aerial insulator images. High Volt..

[35] Ibrahim A., Dalbah A., Abualsaud A., Tariq U., El-Hag A. (2020). Application of Machine Learning to Evaluate Insulator Surface Erosion. IEEE Trans. Instrum. Meas..

[36] RadhaKrishna M.V.V., Venkata Govindh M., Krishna Veni P. (2021). A Review on Image Processing Sensor. J. Phys. Conf. Ser..

[37] Gouveia L.C.P., Choubey B. (2016). Advances on CMOS image sensors. Sens. Rev..

[38] Huang C.C., Huang J.K.T., Lee C.W., Lin C.J. (2012). A CMOS active pixel sensor with light intensity filtering characteristics for image thresholding application. IEEE Sens. J..

[39] Boyle W.S., Smith G.E. (1970). Charge Coupled Semiconductor Devices. Bell Syst. Tech. J..

[40] Litwiller D. (2001). CCD vs. CMOS: Facts and Fiction. Photonics Spectra.

[41] Choi B.S., Kim S.H., Lee J., Seong D., Shin J.K., Lim J., Chang S., Park J., Lee S.J., Kyung C.M. (2018). In-Pixel Aperture CMOS Image Sensor for 2-D and 3-D Imaging. IEEE Sens. J..

[42] De Graaf G., Wolffenbuttel R.F. (2004). Illumination Source Identification Using a CMOS Optical Microsystem. IEEE Trans. Instrum. Meas..

[43] Correia R.G., Pimenta S., Minas G. (2017). CMOS Integrated Photodetectors and Light-to-Frequency Converters for Spectrophotometric Measurements. IEEE Sens. J..

[44] Minas G., De Graaf G., Wolffenbuttel R.F., Correia J.H. (2006). An MCM-based microsystem for colorimetric detection of biomolecules in biological fluids. IEEE Sens. J..

[45] Riba J.-R., Moreno-Eguilaz M., Boizieau M., Ibrayemov T. (2022). Performance Evaluation of Solar-Blind Gas-Filled Sensors to Detect Electrical Discharges for Low-Pressure Aircraft Applications. Sensors.

[46] Sliney D.H. (2016). What is light? The visible spectrum and beyond. Eye.

[47] (2020). Standard Tables for Reference Solar Spectral Irradiances: Direct Normal and Hemispherical on 37° Tilted Surface 2020.

[48] Li Z., Li L., Jiang X., Hu J., Zhang Z., Zhang W. (2016). Effects of Different Factors on Electrical Equipment UV Corona Discharge Detection. Energies.

[49] Ofil Ltd. UV Solar Blind Filters, Imagers and Detectors. http://ofil-ltd.www1.50megs.com/.

[50] Schreiber P., Dang T., Pickenpaugh T., Smith G.A., Gehred P., Litton C.W. (1999). Solar-blind UV region and UV detector development objectives. Optoelectronics’ 99—Integrated Optoelectronic Devices.

[51] Schühle U., Hochedez J.-F. (2013). Solar-blind UV detectors based on wide band gap semiconductors. Observing Photons in Space.

[52] Fossum E.R., Hondongwa D.B. (2014). A review of the pinned photodiode for CCD and CMOS image sensors. IEEE J. Electron Devices Soc..

[53] Garnir H.P., Lefèbvre P.H. (2005). Quantum efficiency of back-illuminated CCD detectors in the VUV region (30–200 nm). Nucl. Instrum. Methods Phys. Res. Sect. B Beam Interact. Mater. Atoms.

[54] De Campos Da Costa J.P., Gounella R.H., Bastos W.B., Longo E., Carmo J.P. (2019). Photovoltaic Sub-Module with Optical Sensor for Angular Measurements of Incident Light. IEEE Sens. J..

[55] Alfaraj N. (2017). A Review of Charge-Coupled Device Image Sensors.

[56] Bosiers J.T., Peters I.M., Draijer C., Theuwissen A. (2006). Technical challenges and recent progress in CCD imagers. Nucl. Instrum. Methods Phys. Res. Sect. A Accel. Spectrometers Detect. Assoc. Equip..

[57] Lee H., Jeong Y.-J., Yoon J., Kang J., Lee S., Shin H., Lee K. (2010). Feasibility study of CCD-based gamma camera. Proc. SPIE Int. Soc. Opt. Eng..

[58] Katikala H.B., Pitchaiah T., Murthy G.R. Low Readout Noise Photodiode based CMOS Image Sensor with High Fill Factor for Biomedical application. Proceedings of the 2022 IEEE Delhi Section Conference (DELCON).

[59] Bigas M., Cabruja E., Forest J., Salvi J. (2006). Review of CMOS image sensors. Microelectron. J..

[60] Mehta S., Patel A., Mehta J. CCD or CMOS Image sensor for photography. Proceedings of the 2015 International Conference on Communications and Signal Processing (ICCSP).

[61] Abe H. Device technologies for high quality and smaller pixel in CCD and CMOS image sensors. Proceedings of the IEDM Technical Digest. IEEE International Electron Devices Meeting.

[62] Stanger L., Wilkes T., Boone N., McGonigle A., Willmott J. (2018). Thermal Imaging Metrology with a Smartphone Sensor. Sensors.

[63] Wilkes T., McGonigle A., Pering T., Taggart A., White B., Bryant R., Willmott J. (2016). Ultraviolet Imaging with Low Cost Smartphone Sensors: Development and Application of a Raspberry Pi-Based UV Camera. Sensors.

[64] Blanc N. (2001). CCD versus CMOS—Has CCD imaging come to an end?. Photogrammetric Week 2001.

[65] Prasad D.S., Reddy B.S. (2017). Digital image processing techniques for estimating power released from the corona discharges. IEEE Trans. Dielectr. Electr. Insul..

[66] Fahrni T., Kuhn M., Sommer P., Wattenhofer R., Welten S. Sundroid: Solar radiation awareness with smartphones. Proceedings of the UbiComp 2011: Ubiquitous Computing, 13th International Conference.

[67] Weiss A., Geisler R., Schwermer T., Yorita D., Henne U., Klein C., Raffel M. (2017). Single-shot pressure-sensitive paint lifetime measurements on fast rotating blades using an optimized double-shutter technique. Exp. Fluids.

[68] Morimoto K., Morimoto K., Charbon E. (2020). High fill-factor miniaturized SPAD arrays with a guard-ring-sharing technique. Opt. Express.

[69] Zang C., Xinjie Z., Shuang H., Lei H., Zhenglong J., Huisheng Y., Zhiwen J. Research on mechanism and ultraviolet imaging of corona discharge of electric device faults. Proceedings of the Conference Record of the 2008 IEEE International Symposium on Electrical Insulation.

[70] Riba J.-R., Bas-Calopa P., Moreno-Eguilaz M. (2022). Analysing the Influence of Geometry and Pressure on Corona Discharges. Eur. J. Phys..

[71] Grum F., Costa L.F. (1976). Spectral emission of corona discharges. Appl. Opt..

[72] Nagi Ł., Kozioł M., Wotzka D. (2019). Analysis of the spectrum of electromagnetic radiation generated by electrical discharges. IET Sci. Meas. Technol..

[73] Nagi Ł., Kozioł M., Zygarlicki J. (2020). Optical Radiation from an Electric Arc at Different Frequencies. Energies.

[74] Garcia J.E., Dyer A.G., Greentree A.D., Spring G., Wilksch P.A. (2013). Linearisation of RGB Camera Responses for Quantitative Image Analysis of Visible and UV Photography: A Comparison of Two Techniques. PLoS ONE.

[75] Liu Y., Lai T., Liu J., Li Y., Yang J., Pei S. 110 kV insulator contamination diagnosis method based on UV imaging. Proceedings of the 2021 IEEE International Conference on Electrical Engineering and Mechatronics Technology (ICEEMT).

[76] Zhou W., Li H., Yi X., Tu J., Yu J. (2011). A criterion for UV detection of AC corona inception in a rod-plane air gap. IEEE Trans. Dielectr. Electr. Insul..

[77] Maistry N. (2015). Investigating the concept of Fraunhofer lines as a potential method to detect corona in the wavelength region 338 nm–405 nm during the day. 19th International Symposium on High Voltage Engineering.

[78] Gounella R.H., Granado T.C., Da Costa J.P.C., Carmo J.P. (2021). Optical Filters for Narrow Band Light Adaptation on Imaging Devices. IEEE J. Sel. Top. Quantum Electron..

[79] TD90 UV Ray Sensitive Camera, Ultraviolet Light Camera Manufacturer. https://www.ulirvisiontech.com/products/uv-see-corona-camera-td90.html.

[80] Corona Camera. https://infraredcameras.com/products/corona-camera.

[81] Uvirco Technologies (Pty) Ltd. The Original Corona Camera Manufacturer. https://www.uvirco.com/.

[82] Williams A.R., Williams G.F. (1993). The invisible image—A tutorial on photography with invisible radiation, Part 1: Introduction and reflected ultraviolet techniques. J. Biol. Photogr..

[83] Ray S.F. (2002). Applied Photographic Optics: Lenses and Optical Systems for Photography, Film, Video, Electronic and Digital Imaging.

[84] Pinnangudi B., Gorur R.S.S., Kroese A.J.J. (2005). Quantification of corona discharges on nonceramic insulators. IEEE Trans. Dielectr. Electr. Insul..

[85] Prasad D.S., Reddy B.S. (2020). Image Saturation as a Tool to Understand the Corona Induced Degradation of Polymeric Insulators. IEEE Trans. Dielectr. Electr. Insul..

[86] Prasad D.S., Reddy B.S. Understanding Corona Discharges using Digital Imaging. Proceedings of the 2018 IEEE International Conference on High Voltage Engineering and Application (ICHVE).

[87] Riba J.-R., Gómez-Pau Á., Moreno-Eguilaz M. (2020). Insulation Failure Quantification Based on the Energy of Digital Images Using Low-Cost Imaging Sensors. Sensors.

[88] Riba J.-R., Abomailek C., Casals-Torrens P., Capelli F. (2018). Simplification and cost reduction of visual corona tests. IET Gener. Transm. Distrib..

[89] Yan D., Zhang Z., Gong H., Ya Y. (2021). Effect of barbed tubular electrode corona discharge EHD flow on submicron particle collection in a wide-type ESP. J. Electrostat..

[90] Pinchuk M.E., Lazukin A.V., Stepanova O.M. (2021). Gas temperature spatial distribution in air corona discharge with plane comb of metal rod electrodes derived from schlieren images. J. Phys. Conf. Ser..

[91] Raulf T., Claudi A., Zander R., Fuchs C. (2020). Evaluation of a Charged Coupled Device Camera for the Detection of Ultraviolet Emissions by Corona Discharges. Lect. Notes Electr. Eng..

[92] Kurimský J., Rajňák M., Cimbala R., Rajnič J., Timko M., Kopčanský P. (2020). Effect of magnetic nanoparticles on partial discharges in transformer oil. J. Magn. Magn. Mater..

[93] Jula N., Alexandru S., Teodor Lucian G. (2016). A new approach related to the corona discharge surveillance. Mircea Cel Batran Nav. Acad. Sci. Bull..

[94] Qian Y., Zhou X., Kong X., Wu Y., Tang X., Zhang Y. The dual-channel ultraviolet/low light CMOS camera using image fusion technique. Proceedings of the Radiation Detectors in Medicine, Industry, and National Security XVIII.

[95] Okino T., Yamahira S., Yamada S., Hirose Y., Odagawa A., Kato Y., Tanaka T. (2018). A Real-Time Ultraviolet Radiation Imaging System Using an Organic Photoconductive Image Sensor. Sensors.

[96] Tith S., Chankow N., Tith S., Chankow N. (2016). Measurement of Gamma-Rays Using Smartphones. Open J. Appl. Sci..

[97] Guo Z., Ye Q., Wang Y., Han M. (2020). Study of the Development of Negative DC Corona Discharges on the Basis of Visible Digital Images. IEEE Trans. Plasma Sci..

[98] Prasad D.S., Reddy B.S., Shakthi Prasad D., Subba Reddy B. (2017). Study of corona degradation of polymeric insulating samples using high dynamic range imaging technique. IEEE Trans. Dielectr. Electr. Insul..

[99] Pi-Camera Spectral Response Curves by Khufkens. http://bluegreen-labs.github.io/raspberry_pi_camera_responses/.

[100] Abomailek C., Riba J.-R., Casals-Torrens P. (2019). Feasibility analysis of reduced-scale visual corona tests in high-voltage laboratories. IET Gener. Transm. Distrib..

[101] Nakamura H., Ohyama R. An image analysis of positive ionic wind velocity under the DC corona discharge in needle-cylinder electrode system. Proceedings of the 2009 IEEE Conference on Electrical Insulation and Dielectric Phenomena.

[102] Zhu Y., Yamashita S., Anami N., Otsubo M., Honda C., Hashimoto Y. Corona discharge phenomenon and behavior of water droplets on the surface of polymer in the AC electric field. Proceedings of the 7th International Conference on Properties and Applications of Dielectric Materials.

[103] Ohyama S., Ohyama R. Ionic wind characteristics of an EHD micro gas pump constructed with needle-ring electrode system. Proceedings of the 2011 Annual Report Conference on Electrical Insulation and Dielectric Phenomena.

[104] Riba J.R., Bogarra S., Gómez-Pau Á., Moreno-Eguilaz M. (2020). Experimental Study of the Corona Performance of Aged Sand-Cast Substation Connectors. Energies.

[105] Riba J.-R., Morosini A., Capelli F. (2018). Comparative Study of AC and Positive and Negative DC Visual Corona for Sphere-Plane Gaps in Atmospheric Air. Energies.

[106] Wang Y., Qian Y., Kong X. (2018). Photon Counting Based on Solar-Blind Ultraviolet Intensified Complementary Metal-Oxide-Semiconductor (ICMOS) for Corona Detection. IEEE Photonics J..

[107] Davari N., Akbarizadeh G., Mashhour E. (2021). Intelligent Diagnosis of Incipient Fault in Power Distribution Lines Based on Corona Detection in UV-Visible Videos. IEEE Trans. Power Deliv..

[108] Zhang Z., Zhang W., Zhang D., Xiao Y., Deng J., Xia G. (2016). Comparison of different characteristic parameters acquired by UV imager in detecting corona discharge. IEEE Trans. Dielectr. Electr. Insul..

[109] Chen T., Yuan S., Li J., Xing S., Zhang H., Dong Y., Chen L., Liu P., Jiao G. (2018). Image registration for a UV–Visible dual-band imaging system. Opt. Lasers Eng..

[110] Cardoso J.A.A., Filho O.O., de Mello D.R. Use of UV cameras for corona tests in high volatge laboratory. Proceedings of the 16th International Symposium on High Voltage Engineering.

[111] Coetzer C., Becker T., West N., Leuschner W. (2021). Investigating an Alternate Detector for Solar-blind Ultraviolet Cameras for High-Voltage Inspection. Proceedings of the 2021 Southern African Universities Power Engineering Conference/Robotics and Mechatronics/Pattern Recognition Association of South Africa (SAUPEC/RobMech/PRASA).

[112] Skubis J., Kozioł M. (2021). Assessment of Partial Discharges in the Air by Application of Corona Camera. Appl. Sci..

[113] Chrzanowski K.B. (2021). Stafiej Measurement of sensitivity of solar blind UV cameras to solar light. Opto-Electronics Rev..

[114] Maistry N., Schutz R.A., Cox E. (2018). The Quantification of Corona Discharges on High Voltage Electrical Equipment in the UV Spectrum using a Corona Camera.

[115] Wang Y., Qian Y. (2020). Evaluation method for the corona discharge of insulator based on convolution neural network with the dual-spectra camera. Opt. Eng..

[116] Wang Y., Qian Y. (2019). Characteristics of the corona discharge of polymer insulators based on solar-blind ultraviolet images. Opt. Eng..

[117] Coetzer C.J., West N. (2022). Radiometric calibration and measurement algorithm for electrical inspection solar-blind ultraviolet cameras. Opto-Electronics Rev..

[118] Lu Y., Hu C., Liu Y., Liu Z., Luo R., Tian D. Study on the Change of Photon Number with Humidity in UV Detection of External Insulation Corona Discharge and Its Correction Method. Proceedings of the 2021 4th International Conference on Energy, Electrical and Power Engineering (CEEPE).

[119] Coetzer C., Djeumen J., West N., Becker T., Walker J., Leuschner W. (2019). Status quo and aspects to consider with ultraviolet optical versus high voltage energy relation investigations. Proceedings of the Fifth Conference on Sensors, MEMS, and Electro-Optic Systems.

[120] Urbaniec I., Fracz P. (2015). Application of UV camera for PD detection on long rod HV insulator. Meas. Autom. Monit..

[121] Liu Y., Yang J., Ma Z., Li Y., Liu J., Lai T. Analysis of the Influence of Various Factors on the Ultraviolet Discharge Detection of 110kV Contaminated Insulators. Proceedings of the 2021 IEEE International Conference on Electrical Engineering and Mechatronics Technology (ICEEMT).

[122] Wang S., Lu F., Chen L., Li H. Study of polymeric insulator external insulation based on UV imaging method. Proceedings of the 2009 IEEE 9th International Conference on the Properties and Applications of Dielectric Materials.

[123] Pang M.-S., Kim W.-J., Kim Y.-S., Kim S.-H. (2012). Corona Discharge Characteristics of Transformer Bushing Model with Contaminnations in Air. J. Korean Inst. Illum. Electr. Install. Eng..

[124] Stranges M.K.W.W., Ul Haq S., Dunn D.G. (2014). Black-out test versus UV camera for corona inspection of HV motor stator endwindings. IEEE Trans. Ind. Appl..

[125] Moore A.J., Schubert M., Rymer N. Technologies and Operations for High Voltage Corona Detection with UAVs. Proceedings of the 2018 IEEE Power & Energy Society General Meeting (PESGM).

[126] da Silva Y.R.S.C., Kuroda R., Sugawa S. (2019). An Optical Filter-Less CMOS Image Sensor with Differential Spectral Response Pixels for Simultaneous UV-Selective and Visible Imaging. Sensors.

[127] Turner J., Igoe D., Parisi A.V., McGonigle A.J., Amar A., Wainwright L. (2020). A review on the ability of smartphones to detect ultraviolet (UV) radiation and their potential to be used in UV research and for public education purposes. Sci. Total Environ..

[128] Igoe D., Parisi A., Carter B. (2013). Characterization of a smartphone camera’s response to ultraviolet A radiation. Photochem. Photobiol..

[129] Igoe D. (2003). Development and Characterisation of a Modified Smartphone Camera for Determining UVA Aerosol Optical Depth. Ph.D. Thesis.

[130] Turner J., Parisi A.V., Igoe D.P., Amar A. (2017). Detection of ultraviolet B radiation with internal smartphone sensors. Instrum. Sci. Technol..

[131] Igoe D.P., Amar A., Parisi A.V., Turner J. (2017). Characterisation of a smartphone image sensor response to direct solar 305nm irradiation at high air masses. Sci. Total Environ..

[132] Igoe D.P., Parisi A.V., Amar A., Downs N.J., Turner J. (2018). Atmospheric total ozone column evaluation with a smartphone image sensor. Int. J. Remote Sens..

[133] Bas-Calopa P., Riba J.R., Moreno-Eguilaz M. (2021). Corona Discharge Characteristics under Variable Frequency and Pressure Environments. Sensors.

[134] Bas-Calopa P., Riba J.-R., Moreno-Eguilaz M. (2022). Measurement of Corona Discharges under Variable Geometry, Frequency and Pressure Environment. Sensors.

[135] Lee B., Farkas D.L., Lee D.H., Cho D., Jang J.E., Hwang J.Y., Kim J., Kim M., Je M., Youn S. (2016). Smartphone-based multispectral imaging: System development and potential for mobile skin diagnosis. Biomed. Opt. Express.

[136] Kwon O.T. (2017). Applications of Smartphone Cameras in Agriculture, Environment, and Food: A review. J. Biosyst. Eng..

